# Validation of a susceptibility, benefits, and barrier scale for mammography screening among Peruvian women: a cross-sectional study

**DOI:** 10.1186/1472-6874-11-54

**Published:** 2011-12-07

**Authors:** Moises A Huaman, Kelly I Kamimura-Nishimura, Mariano Kanamori, Alejandro Siu, Andres G Lescano

**Affiliations:** 1 Universidad Peruana Cayetano Heredia, Lima, Peru; 2 Department of Pediatrics, Bronx-Lebanon Hospital, New York, USA; 3 Department of Epidemiology and Biostatistics, University of Maryland College Park, Maryland, USA; 4 Department of Obstetrics and Gynecology, Hospital Nacional Arzobispo Loayza, Lima, Peru; 5 Department of Parasitology and Public Health Training Program, US Naval Medical Research Unit Six, Lima, Peru

## Abstract

**Background:**

Perceived beliefs about breast cancer and breast cancer screening are important predictors for mammography utilization. This study adapted and validated the Champion's scale in Peru. This scale measures perceived susceptibility for breast cancer and perceived benefits and barriers for mammography.

**Methods:**

A cross-sectional study was conducted among women ages 40 to 65 attending outpatient gynecology services in a public hospital in Peru. A group of experts developed and pre-tested a Spanish version of the Champion's scale to assess its comprehensibility (N = 20). Factor analysis, internal consistency, and test-retest reliability analyses were performed (N = 285). Concurrent validity compared scores from participants who had a mammogram and those who did not have it in the previous 15 months. T-test and multiple regression analysis adjusting for socio-demographic factors, mammography knowledge and other preventive behaviors were performed.

**Results:**

The construct validity and reliability were optimal. Cronbach-Alpha coefficients were 0.75 (susceptibility), 0.72 (benefits) and 0.86 (barriers). Concurrent validity analysis showed an association between barriers and mammography screening use in bivariate (22.3 ± 6.7 vs. 30.2 ± 7.6; p < 0.001) and multiple regression analysis (OR = 0.28, 95% CI = 0.18-0.43). Ages 50-60 years (OR = 2.35, 95% CI = 1.19-4.65), history of prior Papanicolaou test (OR = 3.69, 95% CI = 1.84-7.40), and knowledge about breast cancer and mammography (OR = 3.69, 95% CI = 1.84-7.40) were also independently associated with mammography screening use.

**Conclusion:**

Concurrent validity analysis showed that the Champion's scale has important limitations for assessing perceived susceptibility for breast cancer and perceived benefits for mammography among Peruvian women. There is still a need for developing valid and reliable instruments for measuring perceived beliefs about breast cancer and mammography screening among Peruvian women.

## Background

Breast cancer is the most common malignancy in women worldwide [1]. In Peru, it is estimated that one in every 25 women will develop breast cancer during their lifetimes causing ~1,000 deaths annually just in Lima, the capital city of Peru [2]. In contrast to the decline in mortality associated with the successful implementation of screening programs for early detection of uterine cervical cancer in Peru [2,3], the efforts to reduce breast cancer morbidity and mortality are still insufficient and represent a public health challenge in this country [4].

Mammography screening decreases breast cancer mortality by 16% [5-7]. The American Society of Cancer recommends yearly mammograms starting at age 40 [8]. In Peru, breast cancer screening programs have been implemented following this recommendation. However, prior reports have shown that mammography utilization in Peru is limited. For example, a survey conducted along the northern coast of Peru revealed that only 16% of women older than 40 had received a mammogram at least once in their lifetime [9]. Moreover, less than half of these women were compliant with annual mammography screening [10]. The cost of screening mammograms in Peru varies from 20 to 100 US dollars for uninsured women, who represent half of the country's female population. Mammograms are considered expensive, especially among the poor and very poor groups that have an average monthly income of ~80 dollars and represent about a third of the Peruvian population [11]. Insured and uninsured women in Peru can access mammograms through public and private clinics via providers' referral or through local diagnostic centers that offer screening without a physician's order.

Several factors are associated with the practice of mammography screening [12,13]. In addition to socioeconomic conditions and accessibility to health care, studies have shown that perceived beliefs about breast cancer and breast cancer screening are independent predictors of mammography compliance [14,15]. Thus, perceived benefits and barriers to mammography screening have been explored in diverse ethnic populations using the Health Belief Model (HBM) as a framework [16,17]. Among Hispanic populations, for instance, there is a common belief that cancer is an embarrassing and fatal disease which causes fear of its diagnosis [18,19]. In addition, Hispanic women have a very low perceived susceptibility to breast cancer that leads to poor compliance with screening programs [20]. These beliefs have not been explored in Peru, in part due to lack of instruments locally validated for this aim.

In 1984, Champion validated a scale to measure perceived susceptibility to breast cancer as well as benefits and barriers to breast cancer screening behaviors [21]. A revised version of this scale was published in 1999 showing a significant correlation between mammography compliance and high scores in the susceptibility and benefit subscales; whereas perceived barriers were associated with a lower frequency of mammography utilization. The scale was validated in a population of Caucasians (68%) and African-Americans (30%) with almost no Hispanic or other ethnicities included. The scale accounted for 54% of the variance and showed adequate construct validity and reliability [22].

The aim of our study was to validate a Spanish-translated version of the 1999 revised Champion's scale to measure beliefs about breast cancer and mammography screening in Peruvian women. Ultimately, concurrent validity analysis was performed to determine if the scale was associated with recent mammography screening utilization.

## Methods

### Study design and participants

A cross-sectional study was conducted in women aged 40 to 65 years who attended an outpatient gynecology clinic at Hospital Nacional Arzobispo Loayza, a large tertiary care public hospital in Lima, Peru. It excluded women with prior history of breast cancer or breast surgery, women who sought care due to breast related illness, and those with history of breast cancer in a first degree relative. Eligible women were informed about the study and signed a consent form prior to participation. Sample size was calculated based on expected differences in scale scores between women who had received a screening mammogram in the prior 15 months and those who had not. Thus, at least 253 participants were needed to obtain a difference of at least 0.96 on the susceptibility subscale (the subscale with the smallest expected difference according to original validation study [22]) using alpha error 0.05 and beta error 0.20.

### The scale

The 1999 revised Champion's scale has 19 items distributed in three different dimensions (subscales): susceptibility (3 items), benefits (5 items), and barriers (11 items). Each item was anchored with a five-point Likert scale with response options from 1="strongly disagree" to 5="strongly agree". The scale was originally validated in a cohort of 804 women age 50 or over who were members of a Health Maintenance Organization (HMO) in Indiana, US. Sixty-eight percent were Caucasians and 30% were African-Americans. The other 2% were Asian, Hispanic or Native American [22]. The Champion's scale has been mostly used in the US as a research tool to study the beliefs about breast cancer screening in different populations [23,24] and to develop interventions to improve the utilization of mammography [25]. Multiple adaptations have been validated for other populations around the globe [26-28]. More recently, a Spanish version of the scale was adapted and tested in a cohort of 274 women in Spain [29].

The English [22] and Spanish [29] versions of the Champion's scale were revised by the authors of the study and four bilingual experts. We found minor grammatical and semantic discrepancies in certain items (item 2, 4, 5, 6, 7, 11, 13, 15, 17) that were adapted to local dialect by consensus. A pilot study was conducted in 20 women to ensure understanding of all items. No further modifications to the scale were required as a result of the pilot study. The time for scale completion had a median of 12 minutes.

### Procedures

The scale was administered to all eligible women who accepted to participate in the study. Illiterate participants provided their answers via direct interview. Socio-demographic data was collected from each participant. Participants also answered a questionnaire of knowledge about breast cancer and mammography. This questionnaire was based on an instrument previously validated by Huaman R. in a local intervention for breast cancer prevention in Peru [10].

### Validation of the scale

Construct validity, reliability and concurrent validity of the scale were evaluated using Stata v10.0. To evaluate construct validity we used exploratory factor analysis (EFA) with a viramax rotation. In this analysis we looked for different factors (dimensions) in the scale. Factor extraction was guided by theory and eigenvalues. Items with loading factors above 0.4 were retained in their corresponding factor. Confirmatory factor analysis (CFA) using LISREL [30,31] was conducted to confirm relationships established by the EFA. Thus, a covariance matrix of the 19 items was used as an input in testing the model. The fit of the model to the data was evaluated using Goodness of Fit Index (GFI), which was expected to be close to 0.9 as suggested by Boyd et al [32].

Reliability was evaluated using Cronbach-Alpha and test-retest correlation. Each dimension was expected to have an alpha of at least 0.7. If a dimension's alpha increased more than 0.1 after an item had been eliminated, this item was removed from the scale. Items were also removed if the score of the item and its dimension had a correlation lower than 0.3 after the item was excluded. For test-retest correlation we randomly selected 30 participants to complete the scale 21 to 28 days after the participant had completed the scale for the first time. We compared the test-retest scores for each dimension using Pearson correlation test. We considered that test-retest correlation coefficient should be at least 0.6.

Concurrent validity was evaluated comparing the subscale scores of women who had received a mammogram in the last 15 months with those who had not received a mammogram in the last 15 months, using the t-student test. Finally, we used logistic regression models to adjust the association between the subscale scores and mammogram utilization. The three subscale scores were entered into the model as independent factors. For this, we generated scores from each subscale with the Stata command "predict". The model was adjusted by socio-economic variables, data collection methodology, other preventive behaviors, and knowledge about breast cancer and mammography. The variables that were included in the final model were: perceived susceptibility score, perceived benefits score, perceived barriers score, age (40-to-50, 50-60, 60-to-65 years), region of birth (coast, highlands, jungle), region of current residence (coast, highlands, jungle), formal education (none/elementary, high school, university), the method of data collection (self-administered questionnaire vs. direct interview), the history of Papanicolaou test in the last 15 months, the history of breast self-exams in the last 6 months. We report odds ratios (OR) at 95% confidence interval. P-values less than 0.05 were considered as statistically significant.

### Ethical aspects

This study was approved by the Research Unit of Hospital Nacional Arzobispo Loayza and the Ethics Committee of Universidad Peruana Cayetano Heredia. Personnel from the US Naval Medical Research Unit 6 (NAMRU-6) in Peru helped with data analysis and manuscript edition.

## Results

Between May and June 2008, 296 women completed the scale. Two hundred eighty-five women (96.3%) provided complete data and were included in the analysis, 37.2% had received a screening mammogram in the prior 15 months, and 57.2% reported to have had a screening mammogram at least once during their lifetime. The socio-demographic characteristics of the participants are shown in table 1.

**Table 1:** Participants' socio-demographic characteristics

Characteristics	n	%
Age (media ± SD^a^, 50.2 ± 6.8)		
40 to 50 years	145	50.8
50 to 60 years	106	37.2
60 to 65 years	34	11.9
Region of current residence		
Lima	257	90.4
Coast (other than Lima)	269	94.4
Highlands	15	5.2
Jungle	1	0.4
Level of formal education		
None/incomplete elementary school	41	14.6
Completed elementary school	38	13.6
Incomplete high school	52	18.6
Completed high school	84	30.0
Incomplete university education	28	10.0
Completed university education	37	13.2
Mammogram in the prior 15 months		
Yes	106	37.2
No	179	62.8
Breast self-exam in the prior 6 months		
Yes	201	71.0
No	84	29.0
Papanicolaou test in the prior 15 months		
Yes	191	68.0
No	94	32.0
Scores for each scale dimension^b^	(media ± SD)	
Susceptibilities	9.5 ± 2.7	
Benefits	18.9 ± 3.6	
Barriers	27.3 ± 8.2	

^a ^SD = standard deviation

^b ^possible ranges of scores: susceptibilities (3-15), benefits (5-25), barriers (11-55)

In the EFA we identified three dimensions (subscales) that overall accounted for 48% of the variance. Each of the three dimensions extracted from the EFA had eigenvalues higher than 1 and corresponded to the dimensions of barriers, benefits and susceptibilities of the 1999 revised Champion's scale (table 2). Confirmatory factor analysis supported the results of EFA. The Goodness of Fit Index for these data was 0.89.

**Table 2:** Results of exploratory factor analysis with viramax rotation

Items	Factor 1	Factor 2	Factor 3
SUS1			0.79
SUS2			0.81
SUS3			0.80
BEN1		0.50	
BEN2		0.81	
BEN3		0.63	
BEN4		0.76	
BEN5		0.75	
BAR1	0.65		
BAR2	0.70		
BAR3	0.65		
BAR4	0.65		
BAR5	0.61		
BAR6	0.64		
BAR7	0.65		
BAR8	0.62		
BAR9	0.64		
BAR10	0.60		
BAR11	0.66		
*Eigenvalue*	4.61	2.56	2.1

SUS = susceptibilities; BEN = benefits; BAR = barriers.

All the items had factor loadings above 0.4 in their corresponding dimensions as shown in table 2. Factor 1 which corresponds to perceived barriers had items with loadings between 0.60 and 0.70. Factor 2 which corresponds to perceived benefits had items with loadings between 0.50 and 0.76. Factor 3 which corresponds to perceived susceptibility had items with loading factors between 0.79 and 0.81. After loading in their primary dimension, none of the items had factor loadings above 0.3 in any other dimension which indicates that the items did not overlap dimensions. Standardized factor loadings in CFA were also above 0.4 for each item in its corresponding dimension.

In terms of reliability (table 3), the susceptibility dimension had an alpha of 0.75 with no increase greater than 0.1 after eliminating any items. The item-to-dimension scores correlation was between 0.56 and 0.63. The benefits dimension had an alpha of 0.72 with no increase greater than 0.1 after eliminating any items. The item-to-dimension scores correlation was between 0.31 and 0.62. Perceived barriers had an alpha of 0.86. Item-to-dimension scores correlation was high, between 0.84 and 0.85. Test-retest scores for the three dimensions had Pearson correlation coefficients higher than 0.6 (table 3).

**Table 3:** Reliability of the scale (n = 30 for test-retest correlation coefficient)

ITEM	Item-total correlation	Cronbach Alpha	Cronbach Alpha if item is eliminated	Test-retest correlation coefficient
SUS1	0.56		0.69	
SUS2	0.62		0.62	
SUS3	0.56		0.79	
Total susceptibility		0.75		0.87; p < 0.001
BEN1	0.31		0.76	
BEN2	0.62		0.63	
BEN3	0.43		0.70	
BEN4	0.58		0.64	
BEN5	0.55		0.65	
Total benefits		0.72		0.61; p < 0.001
BAR1	0.57		0.85	
BAR2	0.63		0.84	
BAR3	0.57		0.84	
BAR4	0.55		0.85	
BAR5	0.52		0.85	
BAR6	0.56		0.85	
BAR7	0.57		0.85	
BAR8	0.55		0.85	
BAR9	0.54		0.85	
BAR10	0.51		0.85	
BAR11	0.55		0.85	
Total barriers		0.86		0.94; p < 0.001

The results of bivariate analyses exploring the association between subscales scores and mammography screening in the prior 15 months are shown in table 4. Perceived barriers had significantly lower scores among women who had received a mammogram in the prior 15 months (p < 0.001). In addition, lower barrier scores were found among those with university degrees in comparison to those with high school (p < 0.001) and elementary school/no formal education (p < 0.001). Perceived benefits and susceptibility scores were not associated with mammography screening, formal education level, or age.

**Table 4:** Scores for each scale dimension by mammography use, age and education.

	Susceptibility^a^	Benefits^a^	Barriers^a^
Screening mammogram			
Yes	9.2 ± 2.7	19.2 ± 3.5	22.3 ± 6.7
No	9.8 ± 2.6	18.9 ± 3.7	30.2 ± 7.6
	p = 0.071^b^	p = 0.565^b^	p=<0.001^b^
Age			
40 to 50 years	9.8 ± 2.8	19.1 ± 4.0	16.9 ± 8.3
50 to 60 years	9.4 ± 2.5	19.0 ± 2.8	28.0 ± 8.0
60 to 65 years	9.4 ± 2.5	18.7 ± 4.1	26.5 ± 9.0
	p = 0.477^c^	p = 0.844^c^	p = 0.4773^c^
Education			
None/elementary school	9.9 ± 2.7	19.0 ± 3.0	30.9 ± 6.6
High school	9.5 ± 2.7	19.1 ± 3.8	27.1 ± 8.4
University degree	9.3 ± 2.7	19.0 ± 3.7	22.6 ± 6.9
	p = 0.348^c^	p = 0.956^c^	p=<0.001^c^

^a ^possible ranges of scores: susceptibilities (3-15), benefits (5-25), barriers (11-55)

^b ^t-student test

^c ^ANOVA test

Multiple logistic regression analysis showed that perceived barriers were strongly associated with mammography screening even after adjusting by all the other variables (OR = 0.28 for each additional point in the score, 95%CI 0.18-0.43; p < 0.001). Other factors associated with mammography screening were age between 50 and 60 years (OR = 2.35, 95%CI 1.19-4.65; p = 0.014), knowledge about breast cancer and screening mammogram (mid tercile, OR = 2.18, CI95% 1.09-4.39; p = 0.028; upper tercile, OR = 2.55, CI95% 1.14-2.73; p = 0.023), and history of Papanicolaou test in the prior 15 months (OR = 3.69, CI95% 1.84-7.40; p < 0.001; table 5).

**Table 5:** Factors associated with screening mammography in logistic regression analysis

	Crude Odds Ratio OR (95% CI)	Adjusted Odds Ratio OR (95% CI)^a^
Scale dimensions		
Susceptibilility	0.88 (0.69-1.13)	0.84 (0.63-1.14)
Benefits	1.13 (0.88-1.46)	1.18 (0.86-1.60)^c^
Barriers	0.29 (0.21-0.41)	0.28 (0.19-0.43)
Age		
40 to 50 years^b^		--
50 to 60 years	1.60 (0.96-2.70)	2.35 (1.19-4.65)
60 to 65 years	0.97 (0.43-2.15)	0.91 (0.33-2.46)
Formal education		
None/elementary school^b^		--
High school	2.07 (1.12-3.80)	1.31 (0.59-2.87)
University education	2.38 (1.17-4.81)	0.91 (0.34-2.43)
Region of birth		
Coast^b^		--
Highlands	0.70 (0.43-1.15)	1.31(0.66-2.60)
Jungle	0.71 (0.21-2.49)	0.53(0.11-2.54)
Region of current residence		
Coast^b^		--
Highlands	0.59 (0.15-1.92)	0.36(0.08-1.67)
Jungle^d^		--
Knowledge		
Lower tercile^b^		--
Middle tercile	2.46 (1.4-4.33)	2.18(1.09-4.39)^c^
Upper tercile	3.28 (1.70-6.32)	2.55(1.14-2.73)^c^
Self breast exam	1.59 (0.92-2.75)	0.88(0.43-1.80)
Papanicolaou test	2.96 (1.67-5.23)	3.69(1.84-7.40)^c^
Data collection methodology		
Self-administered^b^		--
Direct interview	0.78 (0.48-1.26)	0.94(0.47-1.88)

^a ^Odds ratio (OR) adjusted by all the other variables in the model.

^b ^Reference category.

^c ^Statistically significant association.

^d ^Dropped from the model, only one participant was living in Jungle region.

## Discussion

Our findings reveal that a Spanish-adapted version of the Champion's scale applied in Peruvian women had optimal construct validity and reliability. Moreover, the completeness of the scale was high (96.3% of participants answered all the questions) which indicates that this instrument was easy and simple to administer. However, the scale had significant limitations in terms of concurrent validity. Perceived barriers was the only subscale associated with recent mammography screening in both bivariate and multivariate analysis. Neither perceived susceptibilities nor benefits were associated with recent mammography screening.

Based on the health belief model as a conceptual framework, studies have demonstrated that beliefs about breast cancer and mammography screening are important predictors of mammography compliance [15,16]. It follows that a scale exploring these beliefs should be able to distinguish between women who had received a mammogram from those who had not received a mammogram. In the original validation study, the three dimensions of the Champion's scale were associated with screening mammography utilization among women from a US city [22]. Although the scale was successfully adapted and validated in Turkey Jordan and Malaysia [26,27], a Spanish-translated version showed important limitations in terms of predictive validity and construct validity when administered in Spain [29]. In our study, although perceived barriers had similar psychometric properties to the original version of the scale, its other components (susceptibilities and benefits) showed poor concurrent validity. Therefore, we consider that this instrument should not be used among Peruvian women.

An explanation of our findings might be that the Champion's scale does not include beliefs about breast cancer and screening mammography that are particularly important for Peruvian women. Therefore, the content of this scale should be reviewed and modified based on qualitative research including interview and focal group techniques with special emphasis on perceived susceptibilities and benefits. Once negative attributes and misconceptions related to poor mammography compliance are identified, targeted interventions could be implemented to promote breast cancer screening in Peru. As demonstrated in a meta-analysis of Yabroff et al, interventions directed to modify negative beliefs improve mammography utilization in 24%; even more effective than phone calls, letter reminders or interventions based on sociologic theories [33]. It is also possible that differences in demographics between our population and the Champion's cohort could have contributed to our results. In the Champion's study, women were 50 years or more with a mean of 61 and had a high mean educational level (12.5 years). In our study, we included women aged 40 or more with a mean of 50. More than 75% of our participants had less than 11 years of education. Moreover, 49% of our sample were interviewed whereas in the Champion's study the scale was only self-administered as those not able to read or write were excluded. Finally, differences in the study design could have contributed to discrepancies between our study and the Champion's study. The later study evaluated the completion of mammogram screening after the baseline assessment of the scale (predictive validity). Our study assessed the scale and mammography utilization at the same time (concurrent validity). In certain circumstances, having a recently negative mammogram could have reinforced the belief that one is not susceptible to breast cancer or that there was no benefit from having the screening, especially among the younger participants.

Our findings suggest that the Champion's scale should be applied with caution in populations with high proportion of Hispanic women. Although our results might not be generalizable to other Hispanic populations, common beliefs about mammography screening may be shared in the region making possible some extrapolation of these results [16]. Previous investigations have shown that most Hispanic women believe that cancer is a fatal illness which generates fear of cancer diagnosis [18,19]. Other barriers include those directly related to the exam technique such as considering mammogram uncomfortable and embarrassing. In addition, Hispanic women consider that breast cancer is a severe illness; however, their own perceived susceptibility to the illness is very low and find scant benefit in regular mammography screening [20]. Recently, Medina-Shepard et al reported high construct validity and reliability of a similar Spanish-translated version of the Champion's scale in a Hispanic cohort from South Florida; but no concurrent or predictive validity analysis was performed [23]. Borrayo et al. have developed a scale for beliefs about breast cancer and mammography screening for Hispanic women validated in a different US city. Although the scale showed strong construct validity, reliability and predictive validity; it has 6 dimensions and 35 items, which might be a limitation for its application in non-research settings, particularly in resource limited areas with a high proportion of illiterate women who require additional personnel to complete the scale. Further research in scale development for breast cancer screening in our region is needed [34].

Logistic regression analysis allowed us to identify other factors associated with mammography screening. Women aged 50 to 60 years had higher rates of mammogram utilization in comparison to those aged 40 to 50 years. Although age is associated with other preventive behaviors such as uterine cervical cancer screening [12], prior studies have reported inconsistent associations between mammography screening and age [12,35,36]. In our sample, the very poor mammography compliance in younger women might be in part due to lack of knowledge about current screening recommendations. Nevertheless, a proportional increase of mammogram utilization was expected among women above 60 years which was not observed in our data. Investigations exploring awareness of current breast cancer guidelines among health care providers are also recommended to determine if practice guidelines need to be reinforced among health care personnel in these settings. Knowledge about breast cancer and mammograms as well as history of recent Papanicolaou tests were associated with mammography screening which correlates with what was reported in prior studies [35,37]. Future interventions to promote mammography utilization in our region should also address this lack of information about breast cancer and screening methods.

This study had some limitations. The history of having received a screening mammogram in the prior 15 months was established by self-report. Thereby, this might have introduced misclassification bias. Participants had the option to complete a self-administered scale or provide their answers via interview. This dual data collection methodology was needed as almost half of our participants were illiterate. This could have introduced a detection bias among women who were interviewed; however, logistic regression analysis showed no significant association between screening mammogram compliance and data collection methodology. Finally, it is important to consider that several studies have shown that health care accessibility is one of the most important factors influencing mammography utilization [35,37]. Our study population included women who attended a gynecology clinic therefore already bypassed the access barrier. This might explain the relatively high rate of recent mammogram utilization in our group (37%) in comparison to similar reports in Peru [9,10]. Thereby, our study population might not necessarily be a representative sample of Peruvian women. Community-based studies are recommended to explore the beliefs about screening mammogram among those who do not routinely access medical care.

## Conclusions

In summary, although the adapted version of the Champion's scale was simple and had optimal construct validity and reliability; concurrent validity analysis showed that the scale has limitations for assessing perceived susceptibility for breast cancer and perceived benefits for mammography among Peruvian women. Our findings suggest that this scale should not be used among Peruvian women until further content review. Further research to develop alternative instruments based on the health belief model is recommended.

## Competing interests

The authors declare that they have no competing interests.

## Authors' contributions

MAH conceived the study, supervised all aspects of its conduction and wrote the manuscript. KIK assisted with the study design, acquisition of data, analysis and interpretation of data and revised the manuscript. MK, AS and AGL assisted with the study design, analysis and interpretation of data and revised the manuscript. All authors helped to conceptualize ideas, interpret findings and review drafts of the manuscript. All authors read and approved the final manuscript.

## Funding

The participation of Dr. Lescano in this project and publication was sponsored by the training grant NIH/FIC 2D43 TW007393 awarded to NAMRU-6 by the Fogarty International Center of the US National Institutes of Health.

## Disclaimer

The views expressed in this article are those of the authors only and do not necessarily reflect the official policy or position of the Department of the Navy, Department of Defense, nor the U.S. Government.

## Copyright statement

Dr. Lescano is an employee of the U.S. Government. This work was prepared as part of his duties. Title 17 U.S.C. § 105 provides that 'Copyright protection under this title is not available for any work of the United States Government.' Title 17 U.S.C. § 101 defines a U.S. Government work as a work prepared by a military service member or employee of the U.S.Government as part of that person's official duties.

## Pre-publication history

The pre-publication history for this paper can be accessed here:

http://www.biomedcentral.com/1472-6874/11/54/prepub
